# Influence of sipunculan (peanut worm) activity on orifice formation in scleractinian *Heterocyathus* for adaptation to soft substrates

**DOI:** 10.1038/s41598-023-49631-y

**Published:** 2024-04-29

**Authors:** Yuki Tokuda, Shuya Kawakita, Asuka Sentoku, Yoichi Ezaki, Naoki Tanaka, Shotaro Nagasawa, Kazumitsu Nakaguchi, Shuhei Yamaguchi, Yusuke Kondo, Susumu Ohtsuka

**Affiliations:** 1 Faculty of Environmental Studies, Tottori University of Environmental Studies, 1-1-1 Wakabadaikita, Tottori, 689-1111 Japan; 2 Department of Physics and Earth Sciences, University of the Ryukyus, Nishihara, Okinawa 903-0213 Japan; 3 Department of Geosciences, Osaka Metropolitan University, Sumiyoshi-ku, Sugimoto, Osaka, 558-8585 Japan; 4 School of Applied Biological Science, Hiroshima University, 1-4-4 Kagamiyama, Higashihiroshima, Hiroshima 739-8528 Japan; 5 Fisheries Laboratory, Blue Innovation Division, Seto Inland Sea Carbon-Neutral Research Center, Hiroshima University, 5-8-1 Minato-Machi, Takehara, Hiroshima 725-0024 Japan

**Keywords:** Marine biology, Palaeontology, Palaeoecology

## Abstract

Mutualism profoundly affects the morphology and ecological evolution of both hosts and symbionts involved. *Heterocyathus* is a solitary scleractinian coral that lives on soft substrata, and sipunculan worms live symbiotically in the tube-like cavities (orifice) inside the coral skeletons. This habitat provides protection to the sipunculan worms against predators and—owing to the mobility of the worms—prevents the coral from being buried with sediments. The orifice growth is closely related to the symbiont sipunculan worms; however, this has not been previously elucidated. Here, we clarified the growth process of scleractinian coral orifices and the influence of sipunculan activity on this. The orifices were originally formed by rapid accretion deposits. The coral soft tissue enveloping the growth edge of the orifice repeatedly retreated to the outer side due to direct damage to the soft part and/or excessive stress caused by the rubbing of the sipunculan through locomotion, excretion, and feeding behaviour. This resulted in a toppled-domino microskeletal structure appearance and maintenance of the orifice growth. These outcomes demonstrate the first example of the direct influence of symbionts on the skeletal morphogenesis of scleractinian corals. The mutualism between the two organisms is maintained by the beneficial confrontation in forming orifices.

## Introduction

Sedimentation smothers and kills corals within hours to days via microbial processes triggered by sediment organic matter^1^. More than 330 coral species, representing 22% of all scleractinian species, inhabit soft substrates and are vulnerable to sediment burial^2^. *Heterocyathus* is a soft-substrate-dwelling solitary coral that cohabitates with a sipunculan worm inside a hollow cavity within the coral skeleton. This habitat, along with the nematocysts of the coral polyps, provides protection to the sipunculan worms against predators. *Heterocyathus* is towed by worms on soft substrates and can escape to the seafloor even when buried in sediments owing to the mobility of the worms^3^. Thus, mutualism between *Heterocyathus* and sipunculan worms facilitates the survival of *Heterocyathus* on soft substrates^3^. *Heterocyathus* planulae attach to skeletons with hollow structures—gastropod shells, scaphopod shells, and vacant tubes of the serpulid polychaete *Ditrupa—*inhabited by sipunculans or the living scaphopod (*Fissidentalium vernedei*)^3^. The attached corals extend their sclerenchyme on the shell surfaces, leaving open orifices that serve as entry and exit points for the sipunculan worms to move and feed^4^. *Heterocyathus*, when attaching to a gastropod shell inhabited by sipunculans, forms either (1) a lower tubular extension from the original orifice of the gastropod shell with the upper part resembling a chimney-like pore (monoporous type) or (2) an extension of the coiled inhabiting space of sipunculan worms with multiple pores after enveloping the shell in the sclerenchyma (polyporous type)^4^. The earliest fossil record of the partnerships between *Heterocyathus* and symbiotic sipunculans is from the latest Early Cretaceous Albian, specifically *Heterocyathus priscus* Stolarski et al., 2001. This species exhibits a lower tubular extension of the orifice (monoporous type). Conversely, the polyporous type appeared at least from the Miocene (e.g., from Java, Gerth, Yabe, and Eguchi^5,6^). Both the recent species of *Heterocyathus* and its oldest known congeners, such as *H. priscus*, commonly exhibit annulation (growth lines) on the inner wall surfaces of the orifices, suggesting a correlation with the epitheca accretion phases^4^. However, the precise processes involved in orifice and pore formation, which are crucial for establishing symbiotic relationships, have not been fully elucidated. Additionally, parasitic crustaceans belonging to the cryptochirid and ascothoracid groups inhabit colonial corals and reside within pits and/or galls created through modifications of the colonial coral skeletons^7–10^. However, the extent of skeletal modifications caused by symbionts in solitary corals has not been thoroughly investigated. This study aimed to bridge this knowledge gap by providing a comprehensive analysis of microskeletal structures to clarify the formation processes of the coiled orifice extension of *Heterocyathus* spp. Moreover, it represents the first attempt to present evidence of intricate skeletal modifications in scleractinian corals that are symbiotically associated with sipunculans.

## Results

### Macroskeletal morphology of *Heterocyathus* spp.

**Figure 1:**
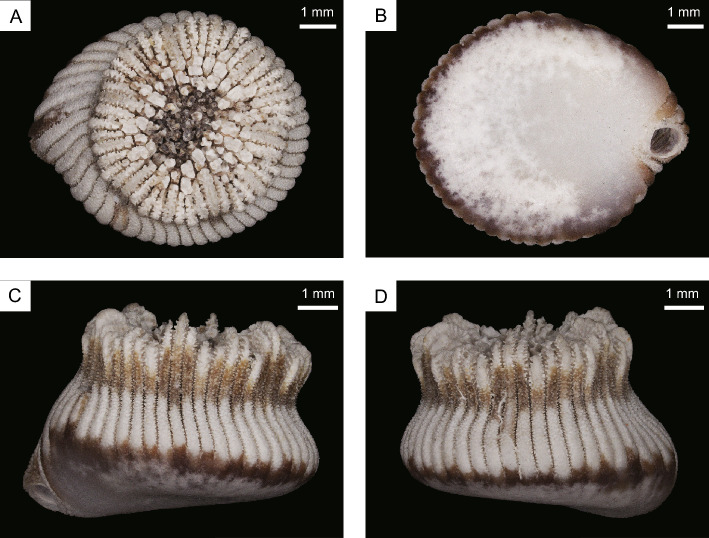
(**A**–**D**) Morphological characteristics of oral (top), bottom, and lateral views of *Heterocyathus* sp. A (polyporous type).

**Figure 2:**
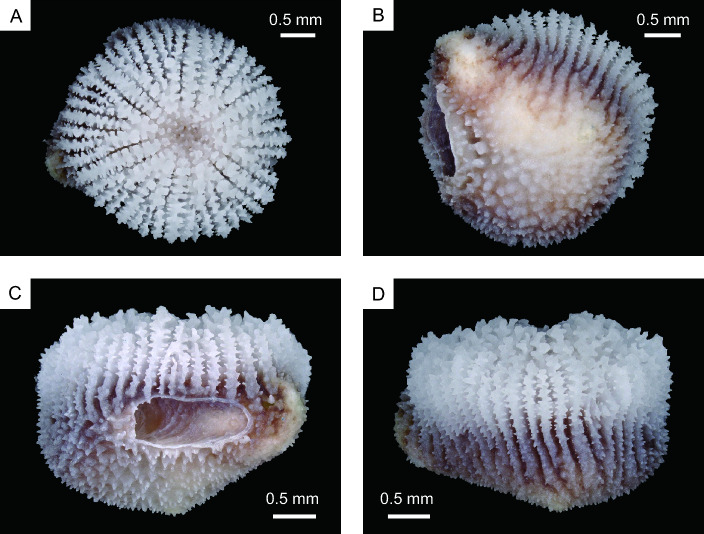
(**A**–**D**) Morphological characteristics of oral (top), bottom, and lateral views of *Heterocyathus* sp. B (polyporous type).

*Heterocyathus* sp. A is a solitary azooxanthellate coral (Fig. 1). The calice is almost circular in cross section with the largest specimen exhibiting a greater calicular diameter of up to 5.9 mm and a height of 5.2 mm. The fully grown corallum of *Heterocyathus* sp. A has a cylindrical shape with a bulging lower part, and its base is slightly flat, featuring an orifice opening at the edge (Fig. 1B). The basal section is thicker on the side where the orifice is located (Fig. 1C,D). The shape of the orifice aditus can vary from circular to elliptical and from 1 to 2 mm in diameter. Additionally, 1–3 pores can be observed on the lateral side of the corallum. Conversely, *Heterocyathus* sp. B is a solitary zooxanthellate coral (Fig. 2) with the largest specimen exhibiting a greater calicular diameter of up to 10.4 mm and a height of 7.3 mm. It has a cylindrical corallum with a slight bulge in the lower part. The base of the corallum is slightly convex in the centre and exhibits costae with distinct costal spines (Fig. 2B–D). The orifice opens on the lateral side of the corallum (Fig. 2B, C), and the shape of the orifice aditus can range from circular to elongated and elliptical with a diameter of 0.5 to 2 mm. The aditus shape is particularly elongated when it is formed along the lateral sides of the gastropod shell (Fig. 2C), and up to seven pores can be observed on the lateral side of the corallum.

### Structure of the orifices of *Heterocyathus* spp.

**Figure 3:**
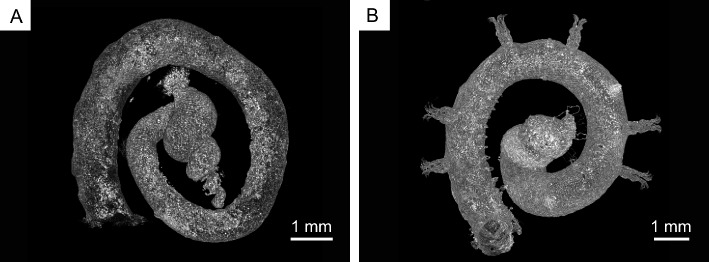
Micro X-ray CT photometry view of the orifice tunnel in *Heterocyathus* sp. A showing a circular to oval single aditus leading to a horizontally curved cylindrical tunnel (not spiral) running nearly parallel to the gastropod shell external surface. (**A**) Tunnel diameter increasing gradually from proximal to distal sides. (**B**) Tunnel diameter decreasing gradually from proximal to distal sides.

Micro-X-ray computed tomography (CT) analysis revealed that the orifice had a single circular to oval aditus, resulting in a horizontally curved cylindrical tunnel (not spiral) running nearly parallel to the external surface of the gastropod shell (Fig. 3). The tunnel diameter gradually decreased or increased from the proximal to distal points, ultimately leading to a tunnel that connects to an aperture linked to the shell whorl (Fig. 3). The calice centres—representing the attachment site of the coral planula—were found to be superimposed on a suture between the body whorl and the penultimate whorl of the gastropod shell in the specimens.

**Figure 4:**
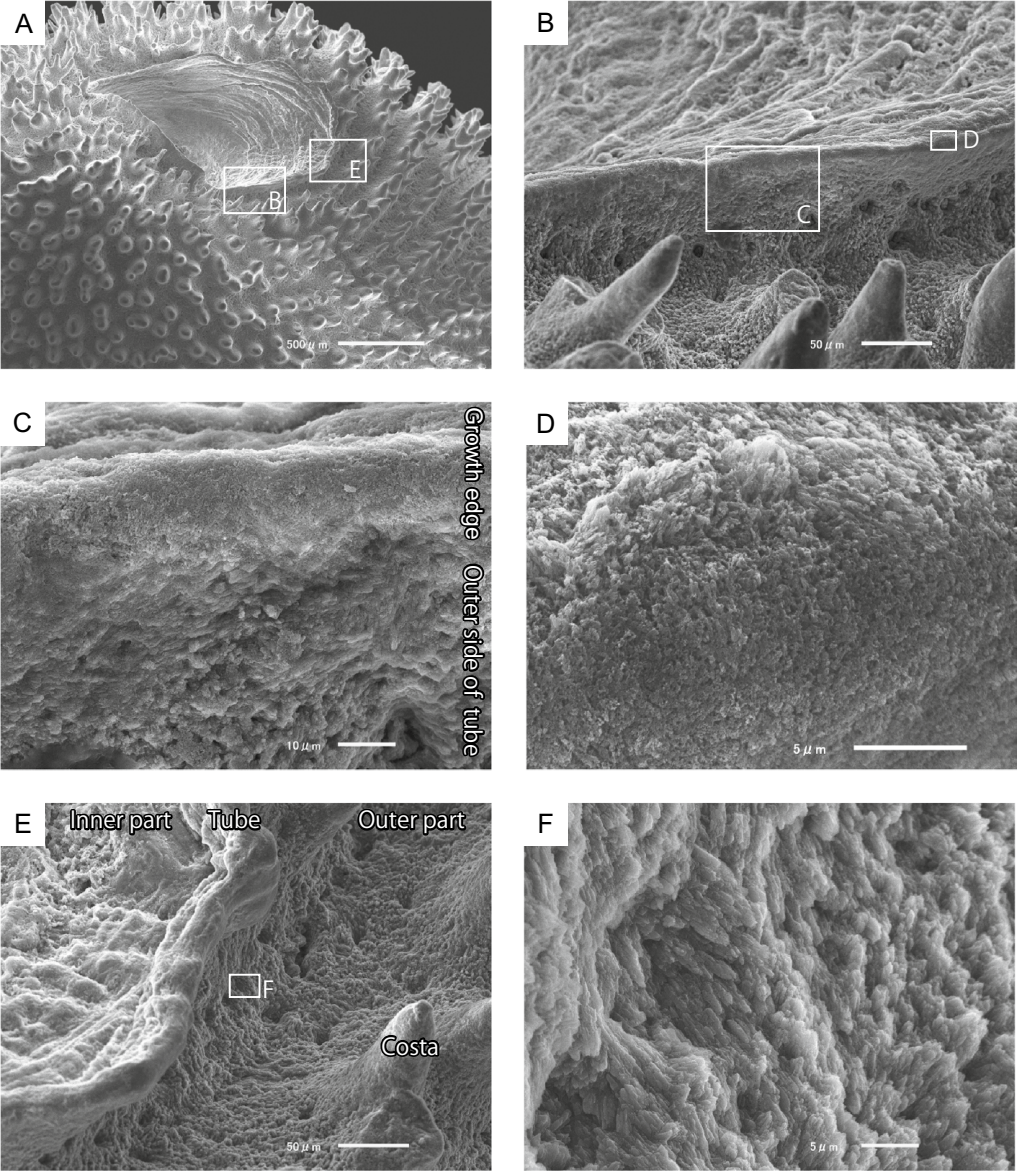
Skeletal microstructures of orifice tube structures. (**A**) Most distal part of the orifice. (**B**) Enlarged tube wall shown in the white square in (**A**). (**C**) Smooth growth edge and rough lower outer side of the tube wall. (**D**) Enlarged growth edge in the white square in (B). (**E**) Enlarged outer side of the tube wall in the white square in (A) showing attachment scars of desmocytes in the lower part of the tube wall. (**F**) Rough surface of the lower outer side of the tube wall comprising irregularly arranged bundles of fibrous crystals.

A short tube structure (10–137 µm high, 7–43 µm wall thickness) protruding from the surrounding part of the corallum was observed in the orifice aditus (Fig. 4A,B). Small fibrous crystals (0.6 µm long, 0.2 µm wide) were observed at the edge of the tube structure (Fig. 4C,D). The uppermost part (approximately 20 µm wide) of the outer surface of the tube structure exhibited a slightly smooth surface (Fig. 4B,C,E). In contrast, the surface of the lower part of the tube was rough and consisted of irregularly arranged bundles of fibrous crystals (1–2.5 µm long, 0.2–0.4 µm wide) that were slightly elongated and perpendicular to the surface, showing attachment scars of desmocytes (Fig. 4E,F).

**Figure 5:**
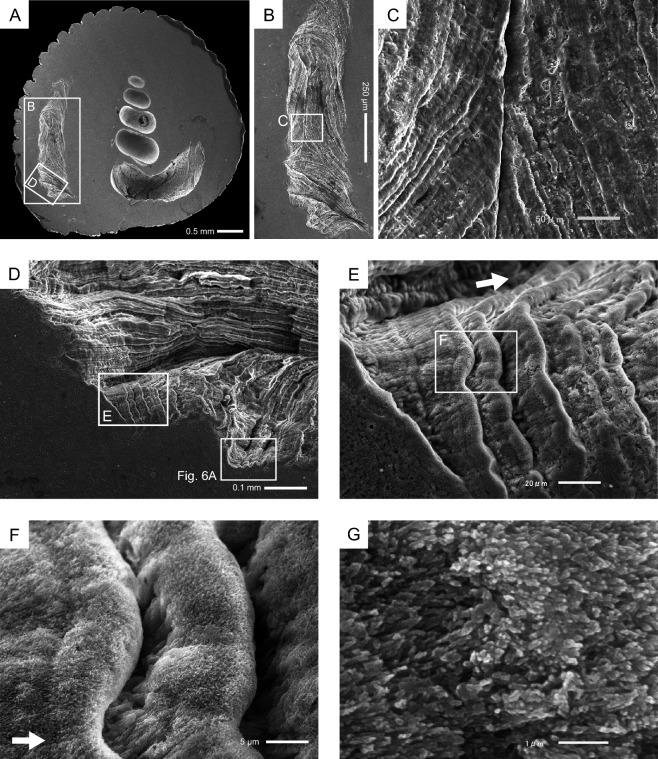
Inner surfaces of *Heterocyathus* sp. B. orifices (**A**) Transverse section of the corallum. (**B**) Distinct growth lines on the lower side of the inner orifice surface. (**C**) Sides of the protruding tube wall partly fused with each other. (**D**) Enlargement of the region shown in the white square in (**A**) showing an undulated tube wall with distinct growth lines. (**E**,**F**) Close-up view of an imbricated growth unit appearing like toppled dominoes. Each unit has a finer groove of growth lines. The white arrow points to the maximum growth direction of the orifice. (**G**) Units comprising extremely fine fibrous crystals.

Distinct growth lines comprising alternating ridges and grooves were observed on the inner surface of the tube structure (Fig. 5). Furthermore, repeated furrows and ridges of growth lines parallel or perpendicular to the maximum growth direction of the orifice tunnel, were observed on the inner orifice surface (Fig. 5B–D). The former type growth lines exhibited successive forward growth (Fig. 5E,F). The latter type growth lines showed that growth edges on both upper and lower sides of the orifice tube structures grew downward and upward and fused with one another to form tube-like hollow structures (Fig. 5B,C). Small fibrous crystals (approximately 0.5 µm long) were observed on the growth line surfaces (Fig. 5G).

**Figure 6:**
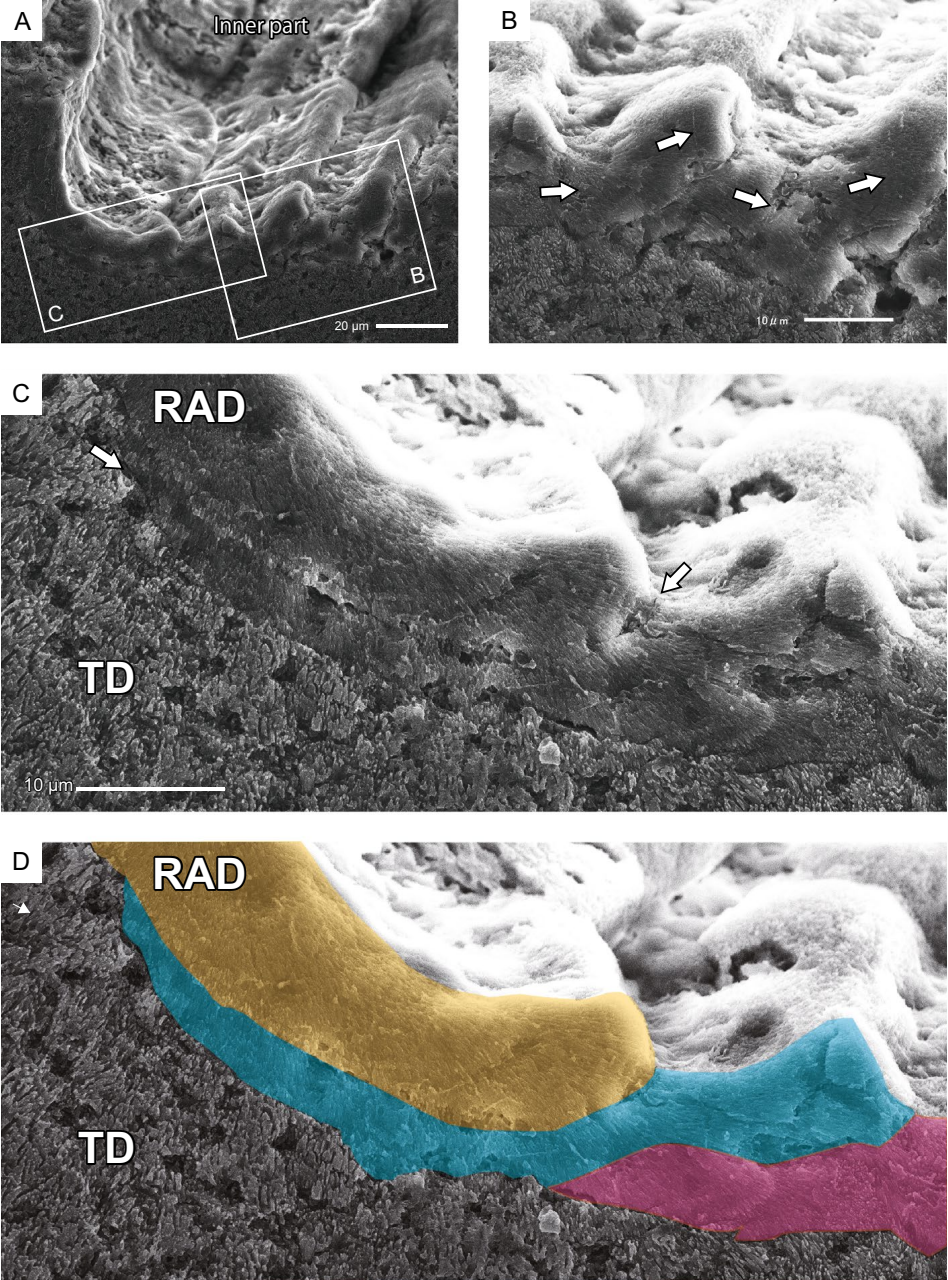
Polished and etched sections of an orifice parallel to the maximum growth direction of *Heterocyathus* sp. B. tunnels (**A**) Close-up view of the concatenated units of tabular-like skeletal structure (shown in the square in Fig. 5D) that comprise rapid accretion deposits (RADs) in the innermost part of the tunnel wall of the orifice with growth lines of the inner orifice surface. (**B**) Growth direction of the tabular-like skeletal units periodically changing inward and outward. The white arrow points to the maximum growth direction of the orifice. (**C**) Enlargement of the region shown in the white square in (A) showing concatenated units of the tabular-like skeletal structures of RADs appearing like toppled dominoes. Only the outer RAD side of the concatenated units were covered with thickening deposits (TDs). (**D**) Pseudo-coloured image of (**C**) showing respective units of tabular-like skeletal structures. Each unit was painted with a different colour.

The polished and etched sections of the orifice, parallel to the maximum growth direction, revealed concatenated units of tabular-like skeletal structures. These structures consisted of rapid accretion deposits (RADs)—containing small fibrous crystals (1–2 µm long, 0.05 µm wide)—with slightly dome-shaped strands (approximately 3–30 μm wide) in the innermost part of the orifice tunnel wall (Fig. 6A,B). Each tabular-like skeletal unit grew slightly inward and thickened, particularly in the distalmost part (i.e., the growth edge). A new unit then regrew from the outer lateral side of the previous unit and along its side (Fig. 6C,D). The concatenated units of tabular-like skeletal structures exhibited an imbricated or toppled-domino appearance (Fig. 6B–D). The distalmost part of the unit corresponded to the tube structure in the orifice aditus. Only the outer sides of the RADs in the concatenated units were covered with thickening deposits (TDs), consisting of fibrous crystals (2–3 µm long, 0.1–0.3 µm wide) (Fig. 6C). The ridges of the growth lines on the inner orifice surface aligned with the thickened distal part of each unit.

**Figure 7:**
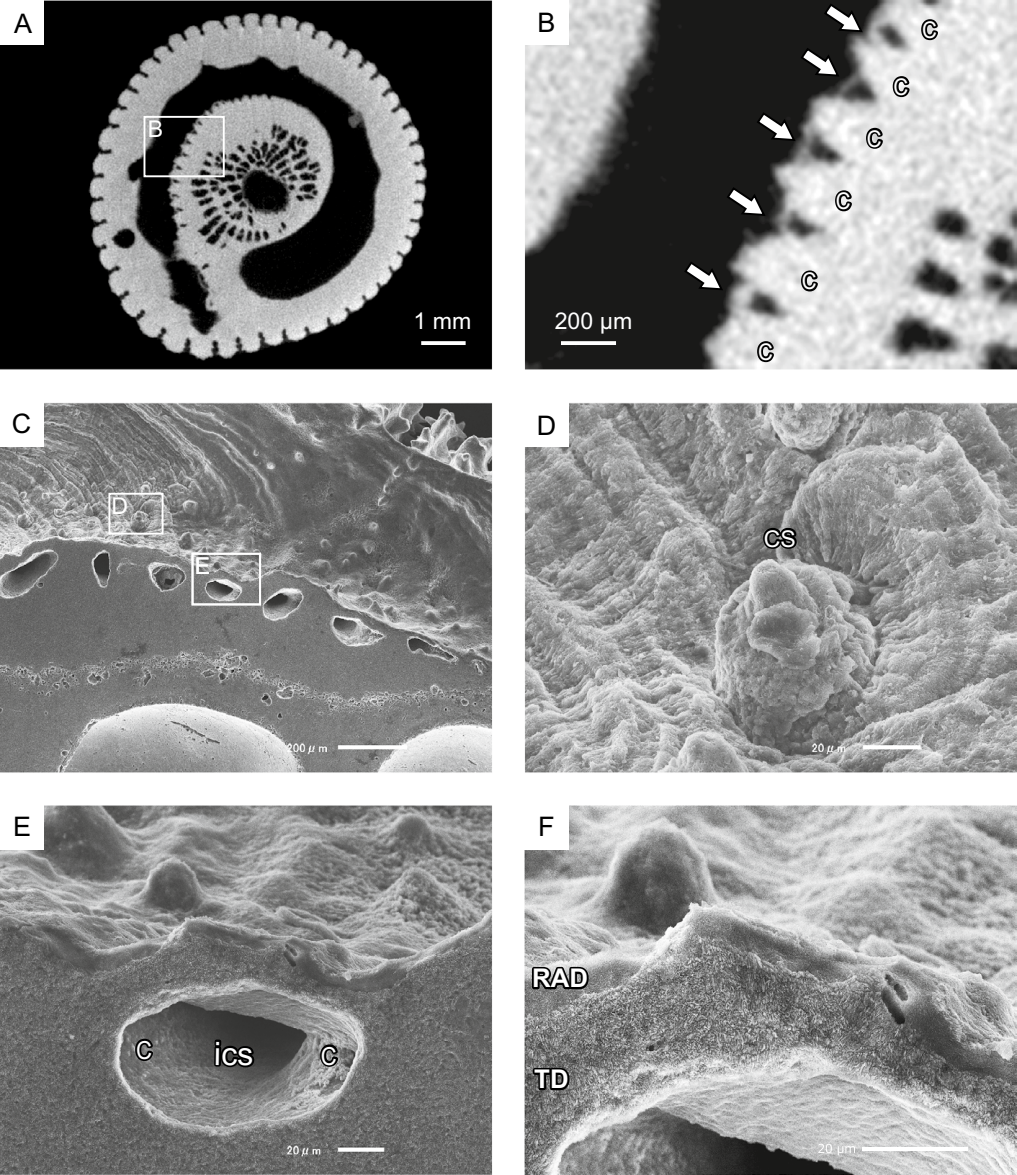
Growth mode of calical side orifices. (**A**,**B**) Micro X-ray CT photography of *Heterocyathus* sp. B. showing thin orifice tube walls (white arrows) covering costae and intercostal spaces. (c) costa. (**C**–**F**) Scanning electron photomicrographs of the calical side of the orifice tunnel. (**C**) Tube walls covering costae and inter costal spaces. (**D**) Costal spine tips piercing through the tube wall. The spines were thinly covered and encircled with the tube walls. (cs) costal spine. (**E**,**F**) Cavities of inter-costal space were observed below the RADs with the TD sheets of tube walls. (ics) inter costal space.

Costae with costal spines and intercostal spaces, located on the inner side of the tunnel, were enveloped by a thin skeleton that contributed to orifice formation (Fig. 7A–C). In some instances, the costal spines penetrated the inner wall of the orifice tunnel (Fig. 7D). These spines were sparsely covered and surrounded by tabular skeletal RAD units, which exhibited distinct growth lines. Additionally, cavities originating from the intercostal spaces or spines were observed beneath the sheet composed of RADs and TDs (Fig. 7E,F). The TDs extended towards the cavities but did not completely fill the space.

**Figure 8:**
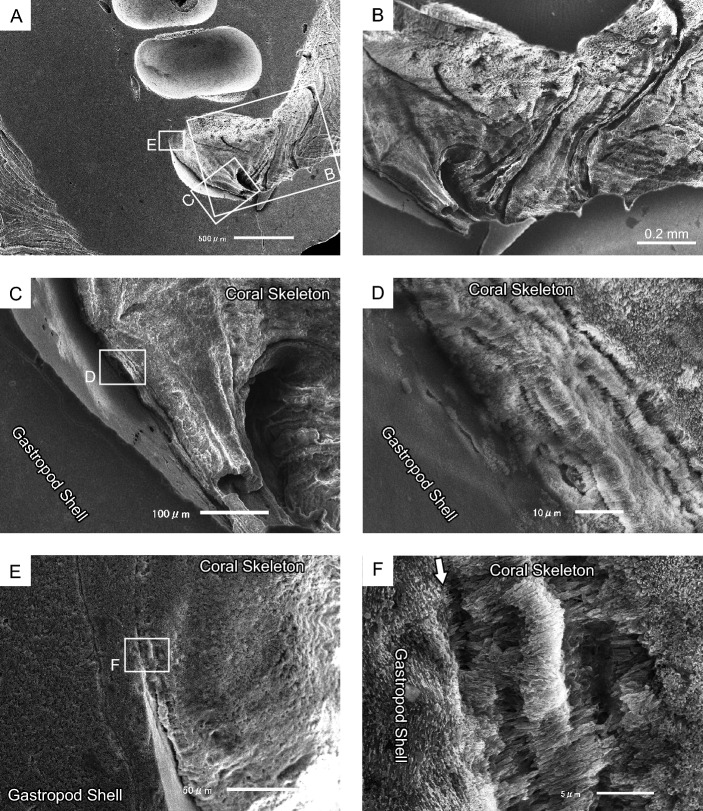
Conjunction part of the aperture of the gastropod shell and the orifice in *Heterocyathus* sp. B. (**A**) Coral skeletal observed on the inner surface of the body whorl of the gastropod shell. (**B**) Growth lines on the inner surface of the early orifice growth part. (**C**–**F**) Boundaries between gastropod shell and coral skeleton. Coral skeletal layer substrates comprising fibrous crystals, elongated and perpendicular to the shell surface at the interface with gastropod shell substrates. (**C**,**D**) Terminal part of the entry of the coral skeleton into the gastropod body whorl. (**E**,**F**) Longitudinal section of the boundary (white arrow) between coral skeleton and gastropod shell.

In addition to the gastropod shell serving as a substrate and orifice, coral skeletal layers were observed on the smooth inner surface of the body whorl (Fig. 8A–D). Each layer (1–5 µm thick) comprised fibrous, elongated crystals that were perpendicular to the shell surface (Fig. 8D–F). These layers were overlaid with RADs containing small fibrous crystals whose growth directions differed from those observed in the lower layers. Furthermore, growth lines were visible on the upper surface of the RAD layers in the proximal region, similar to those observed on the inner orifice surface. These skeletal microstructures are observed in both *Heterocyathus* sp. A and sp. B.

### Soft part arrangement and behaviour of living *Heterocyathus* spp.

**Figure 9:**
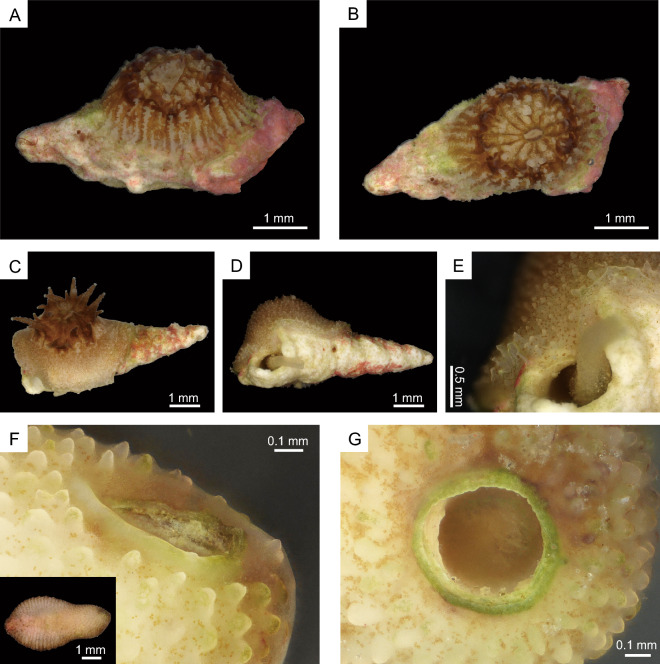
Growth process and soft part distribution of *Heterocyathus* sp. B. (**A**,**B**) Early growth stage of the living part and not an extension on the upper shell side. (**C**–**E**) Living part slightly inserted in the aperture of the gastropod. (**F**,**G**) Growth edge of the orifice tube structure. (**F**) Tube enveloped by the soft part. (**G**) Growth edge 6 days after (**F**), with the soft part only on the outer part of the tube structure.

**Figure 10:**
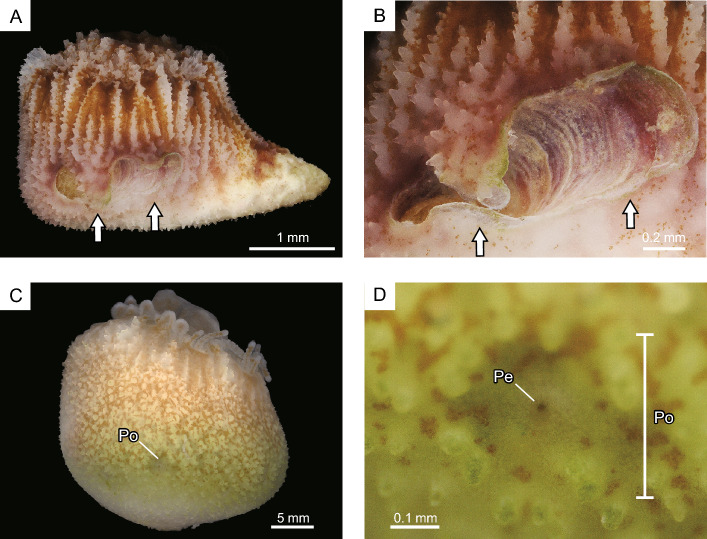
Pore formation of *Heterocyathus* sp. B. (**A**,**B**) Pore formation by a tongue-like growth edge (white arrows) of the upper part of aditus extended to its lower part. (**C**,**D**) Fully developed pore completely covered by coral soft tissue with a small perforation. (Po) pore, (Pe) perforation.

During the early growth stages, the outer soft tissue of *Heterocyathus* sp. B was only present around the calice on the gastropod shell surface (Fig. 9A,B). In subsequent stages, the soft sclerenchymal tissue extended from the upper side of the aperture to the inner whorl of the gastropod shell (Fig. 9C–E). Eventually, the shell surface was completely enveloped by the outer soft tissue, which had rudimentary sclerenchyma and outer extension of costae, preserving the morphology of the gastropod shell within the corallum (Fig. 2B–D). In the orifice aditus, two patterns of soft tissue distribution were observed at the growth edge of the tube structure: (1) complete soft tissue coverage (Fig. 9F) and (2) only on the outer part of the tube structure, without extending to the growth edge and inner orifice surface (Fig. 9G). The elongated elliptical aditus displayed tongue-like protrusions on the middle part of the upper side, growing towards the lower side of the aditus and fusing with each other (Fig. 10A,B). Furthermore, the aperture in the proximal part of the aditus, separated by a tongue-like growth, remained as pores. Fully developed pores were completely covered with coral soft tissue with a small perforation in the pore centre (Fig. 10C,D).

**Figure 11:**
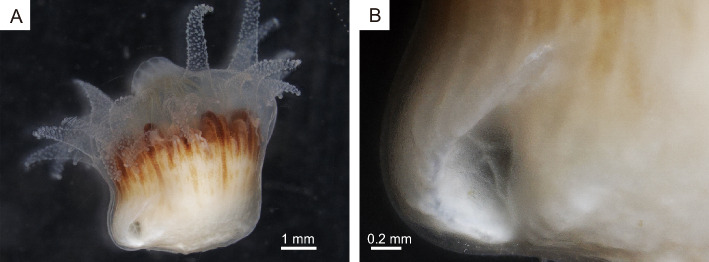
Orifice aditus of *Heterocyathus* sp. A lacking sipunculan worms. (**A**) Lateral view of the corallum with an orifice. (**B**) Enlarged view of the orifice aditus completely covered with the soft part.

**Figure 12:**
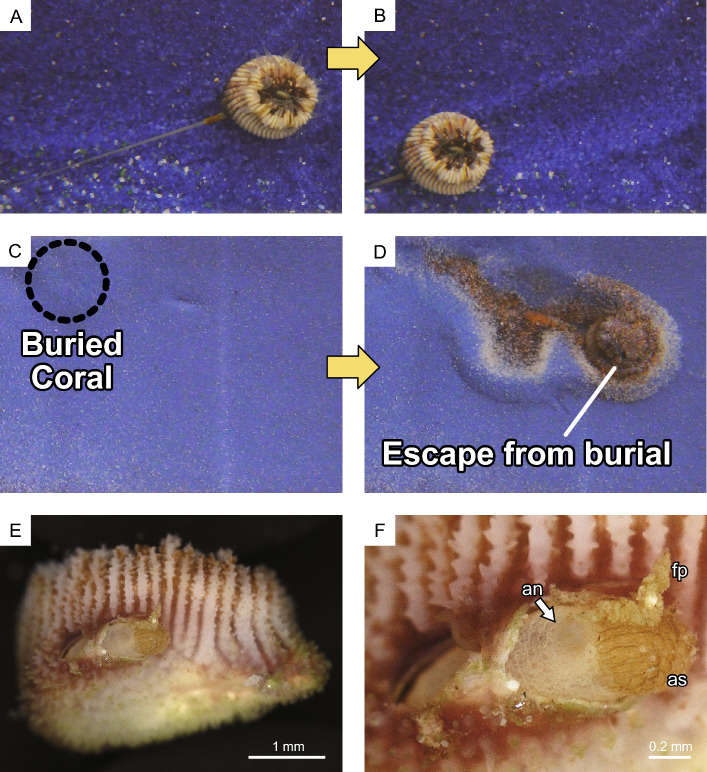
Behaviour of sipunculan worms. (**A**,**B**) Worm-towing *Heterocyathus* sp. B. (**C**,**D**) *Heterocyathus*, buried by the sandy sediment, escaping from burial by sipunculan towing. (**E**,**F**) Excretion of sipunculan by pushing its anus out of the orifice aditus. (an) anus, (as) anal shield, and (fp) faecal pellet.

The orifice aditus of *Heterocyathus* sp. A without sipunculan worms—which were probably dead or had escaped—was covered by a membrane consisting of coral soft tissue extending from the outer part of the tube (Fig. 11). In the experimental tank, the sipunculan extended its introvert into the surrounding substrate and towed *Heterocyathus* spp. (Fig. 12A,B). Additionally, corals that were completely buried in sandy sediment were able to escape with the assistance of sipunculan towing (Fig. 12C,D). The anus of the inhabiting sipunculan was located at the base of the introvert. Excretion in the sipunculan occurred by protruding the anus out of the orifice aditus where faecal pellets were deposited (Fig. 12E,F; Supplementary Movie S1).

## Discussion

### Orifice and pore formation in *Heterocyathus*

**Figure 13:**
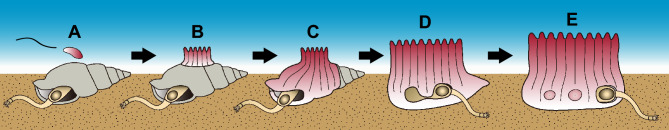
Schematic diagram depicting the growth process of *Heterocyathus* (polyporous type). (**A,B**) The *Heterocyathus* planula attaches to the gastropod shell inhabited by sipunculans. (**C**) The soft sclerenchymal part of the corals spreads over the shell surface up to the inner whorl of the gastropod, and the orifice starts growing from inside the shell. (**D**) The formation of a pore occurs through the fusion of tongue-like prolonged growth edges in the middle part of the upper side of the elongated elliptical aditus. (**E**) The fully developed pore is completely covered by coral soft tissue with a small perforation in the pore centre.

Micro-focus X-ray CT analysis indicated that the *Heterocyathus* planula was attached to a suture between the body whorl and penultimate whorl. This was the posterior surface of the shell opposite the aperture of the gastropod shell, which harboured the sipunculans that faced the substrate side. Therefore, the planulae could attach itself only to the upper side of the shell (Fig. 13A,B). The soft sclerenchymal part of the corals spread over the shell surface up to the inner whorl of the gastropod, and the orifice started growing from inside the shell (Figs. 8A,B, 9C–E, 13C). Attachment of the planula near the aperture may be advantageous for the early initiation of orifice formation. The orifice morphology was horizontally curved, apart from a spiral, cylindrical tunnel surrounding the gastropod shell surface and/or the costae of the side of the corallum (Fig. 3). Moreover, the costae and intercostal spaces were covered with the inner sides of the orifice wall (Figs. 7, 10A,B). Because orifices grew simultaneously with the coralla or around gastropod shells, their external morphologies were regulated by those of the corals and/or shells (Figs. 3, 10A). The orifices with the shortest length were formed with the skeletal material in an energy-saving process.

The diameter of the orifice tunnel gradually decreased or increased from the proximal to the distal sides (Fig. 3). Moreover, the inner sides of the orifice were essentially bioeroded and microborings appeared on the surface (Figs. 5C, 6A). Skeletal erosion is sometimes intense, particularly in the proximal part of the orifice surface (Fig. 8A,B). Herrán et al.^11^ reported erosions in the inner side of an orifice of sipunculan worm*-*harbouring *Heteropsammia*. Sipunculans enlarge the tunnels of their burrows using anal and posterior shields as the animal moves back and forth within its burrow^12^ or by decalcification secretions from the epidermal glands of the *Aspidosiphon*^13,14^. The observed intense erosion, leading to the diameter patterns decreasing toward the distal part of the orifice, could be attributed to the activities of both microboring organisms and sipunculan worms.

Additionally, pore formation in *Heterocyathus—*previously reported by Sluiter^14^ (p. 24)—occurs through the fusion of tongue-like prolonged growth edges in the middle part of the upper side of the elongated elliptical aditus, extending towards the lower side of the aditus (Figs. 10A,B, 13D). This process results in the formation of an opening in the proximal part of the aditus, which remains as a pore. The fully developed pores are completely covered by coral soft tissue with a small perforation (Figs. 10C,D, 13E) through which water circulation in the orifice probably occurs.

The main speculation regarding pore function is water circulation within the sipunculan-inhabited tubes, which has been proposed for both the upper pore (monoporous type) and series of pores (polyporous type)^12,15^, or sipunculan defaecation through pores close to the orifice (polyporous type)^14,16^. This study showed that the fully developed pores were completely covered by coral soft tissue. Moreover, sipunculans excreted by pushing the anus out of the orifice aditus, and not through the pores, even if they were close to the orifice (Fig. 12E,F; Supplementary Movie S1). Future studies should focus on describing the pore functions more comprehensively.

### Microskeletal structures of the orifice

Growth lines were observed on the inner surface of the orifice tunnel (Fig. 5). These growth lines have also been reported for *H. priscus* Stolarski et al., 2001 and *H. mai* Cheng, 1971 (= *H. alternatus* Verrill, 1866). This formation was considered to be related to the accretion phases of the epitheca based on their superficial morphological similarity^4^. However, the skeletal microstructure of this orifice has not yet been elucidated. This study showed that the growth lines were formed by outer accretions of the tabular-like skeletal unit that was thickened, especially at the distalmost part (Fig. 6), which corresponded to the tube structure in the orifice aditus (Fig. 4). This unit comprised small crystals with finely domed or curved growth lines, and the outer side of the unit was covered with large fibrous crystals (Fig. 6C,D). The skeletal layers corresponded to the RADs and TDs (sensu^13^). At the microstructural level, scleractinian skeletons comprised two representative components, RADs and TDs^17^. Although RADs are typical of the growth edges of septa and walls, the *Heterocyathus* orifices were originally formed by RADs. Interestingly, the ability to secrete RADs—unique to scleractinian skeletons—is appropriate for orifice formation, essential for the symbiosis of sipunculan worms and *Heterocyathus*. Furthermore, this homologous microskeletal relationship suggests that orifice formation does not require any special evolutionary innovation in corals.

**Figure 14:**
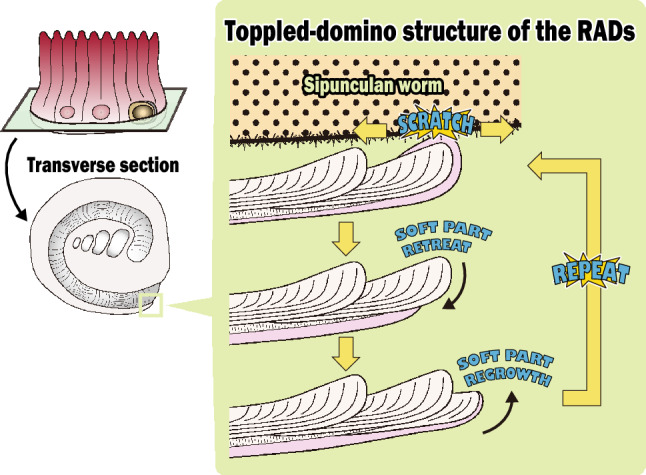
Schematic diagram depicting the growth process of the toppled-domino structures of the RADs in the orifice of *Heterocyathus*.

When considering only one unit with dome-shaped strands, the skeletal structure of the *Heterocyathus* orifice was similar to the RADs of solitary coral walls, such as in *Flabellum* (*Flabellum*) and *Balanophyllia* epitheca, which are composed alternatively of microcrystalline and fibrous parts^18,19^. However, unlike *Heterocyathus*, these corals exhibited continuous RAD layers without toppled-domino structures. While the outer surface of the corallum of *Heterocyathus* sp. B was completely enveloped by soft tissue, the inner orifice surfaces were not, except for the growth edge (Fig. 9F,G). These findings are consistent with the absence of TDs on the inner side of the tabular-like skeletal units. In contrast, soft tissue partially covered the growth edge of the tube structure (Fig. 9F,G). Polypal contraction and rejuvenescence occur only due to polypal damage and/or stress^20–22^. The peripheral sclerenchymal tissue of the polyp often shrinks during the process. In *Heterocyathus*, the retreat of the enveloped inner soft part of the orifice tube structure to the outer side could have been caused by damage or stress only to the soft part on the inner sides of the tube, as other parts of the corallum did not exhibit polypal contraction or rejuvenescence. Furthermore, the expression of different genes in the organic matrix of the calicoblastic ectodermal cells controls the formation of skeletal microstructures in RADs and TDs^23,24^. TDs formed only on the outer surface of the tube structure of *Heterocyathus* (Fig. 6C,D). Calicoblastic cells, responsible for the formation of RADs in the concatenated units of tabular-like skeletal structures, must be exclusively distributed in the most peripheral soft part (i.e., the growth edge). The new unit with a tabular-like skeletal structure regrew from the outer lateral part of the previous unit along its sides (Fig. 6C,D). This toppled-domino structure suggests that the skeletal part of the RADs shifted to the outer part of the last unit, possibly due to the shift or shrinkage of soft tissue containing the RAD-forming calicoblastic cells. The two observed patterns of soft tissue distribution at the growth edge of the tube structure in the same living individuals imply such soft tissue shifting (Fig. 9F,G). The lower part of the outer surface of the tube structure exhibited desmocyte attachment scars, which anchored the soft tissue to the skeletons (Fig. 4B,E,F). The soft part likely receded from the inside to the outside of the growth edge of the tube, possibly terminating at the desmocytes where it anchored to the soft tissue. The presence of short tabular-like units (10–50 µm wide) suggests that the soft part retired frequently, with each unit being formed repeatedly for a short duration, resulting in the toppled-dominoes pattern (Fig. 6). Common rejuvenescence or polypal contraction and recovery caused by any environmental change (e.g., warming) is an extremely slow phenomenon, occurring for at least several months or years^22^. The unusually rapid retreat of the soft tissue in *Heterocyathus* may be attributed to the activity of sipunculan worms, which affect the condition of the soft tissue in specific microspaces of the inner surface of the orifice tube. Notably, the distal part of the sipunculan introvert often possesses hooks^25,26^. Additionally, in *Aspidosiphon*, the epidermis forms specialized structures, such as an anal shield with a hardened, horny structure^25,26^. In our observations, we noticed that *Aspidosiphon* towed *Heterocyathus* by extending and contracting its introvert in the experimental tank (Fig. 12A–D). Furthermore, sipunculans excreted by pushing the anus and anal shield out of the orifice aditus (Fig. 12E,F). Consequently, sipunculans frequently and repeatedly moved back and forth through the orifice aditus, rubbing their hardened anal shield and hooking against the soft part that enveloped the growth edge of the tube structure of *Heterocyathus*. Consequently, this soft part could retreat to the outer side of the tube structure due to direct damage or excessive stress caused by the sipunculan rubbing activity. The orifice aditus of *Heterocyathus* that did not host sipunculan worms was covered with a coral soft part (Fig. 11), also reported by Fisk^27^. These findings strongly suggest that the activity of sipunculan worms prevents corals from sealing their orifices. The retreated soft part, which formed the RADs of the tabular-like skeletal unit on the outer surface of the tube structure, was not directly rubbed by the sipunculan worms because the unit grew along the outer lateral side of the last unit as a shield. Subsequently, this unit overgrew the previous unit, and the exposed soft part of the RAD growth edge of the unit was directly affected by the sipunculan worm activity. Thus, the toppled-domino structure of the RADs in the orifice—along with the growth lines on the inner surface—was formed not only through coral biomineralization but also through sipunculan worm control of the coral soft part at the growth edge of the orifice, thereby maintaining its hollow tunnel structure (Fig. 14). The presence of growth lines, a consequence of the continuously forming toppled-domino structure of the RADs, indicates the mutualistic relationship between corals and sipunculans in orifice formation. Furthermore, the discovery of growth lines on the inner surface of the orifice in the oldest fossil, *Heterocyathus priscus*^4^, suggests the presence of a mutual relationship in the early evolution of *Heterocyathus* since the Cretaceous period.

This study emphasizes the influence of sipunculan activity on orifice formation, which is obligatory for maintaining the symbiosis between *Heterocyathus* and sipunculan worms. These findings provide important clues as to why the symbiotic relationship between corals and sipunculan worms occurs repeatedly in scleractinian corals.

## Methods

We examined 16 skeletal and three living specimens of the azooxanthellate *Heterocyathus* sp. A, collected from a depth of 80 m off the Kerama Islands, Okinawa Prefecture, by R/V Toyoshio-Maru. Additionally, six living specimens of the zooxanthellate *Heterocyathus* sp. B were collected at a depth of 10 m in Kin Bay and 60 m off Sesoko Island, Okinawa Prefecture. Among these, the living specimens from the Kerama Islands were selected for soft part observations and were maintained for more than 2 years in an acrylic tank (150 cm long, 60 cm wide, and 60 cm high) equipped with a mechanical particle filter and a thermostat-controlled cooling system (ZRC-400B; Zensui, Japan). The specimens from Kin Bay and off Sesoko Island were maintained for more than 5 months in another tank (130 cm long, 100 cm wide, and 30 cm high; PURA FUNE 140, RISU KOGYO, Japan) with a filtration system (Eheim external filter professional 5e 2076, Eheim, Germany). The tanks were filled with saline groundwater obtained from a depth of approximately 50 m on the grounds of the Tottori Prefectural Farming Fisheries Centre. The water temperature in the two tanks was maintained at 19 and 25 °C, respectively, to replicate the temperatures observed in natural coral habitats. Initially, the corals were cultured in darkness and were fed frozen copepods twice a week. Subsequently, overhead LED lamps with a power of 24 W (LM24DCII/UVS, B.R.S., Japan) were installed to provide lighting for a photoperiod of 12:12 h, and the corals were fed once a week. The bottom of the tank, where the corals were placed, was covered with medium-to-fine sand substrates.

Macro-to-meso-skeletal features were examined using a digital microscope (VHX-7100; Keyence, Japan). To ensure stable observation of live corals, focus stacking micrography was performed using a stage mount to minimize water surface fluctuations caused by head movements. The specimens were positioned on a 4 cm square anti-vibration rubber pad within a high-glass Petri dish (6.4 cm diameter, 2 cm height) (FS–60, As One, Japan) placed inside a small flat acrylic case (15 cm long, 10 cm wide, and 4 cm high; Seria, Japan). The water depth in the acrylic case was maintained at 3 cm. Additionally, a glass-bottom Petri dish (7.5 cm diameter, 2 cm height) (FS–70, As One, Japan) without water was positioned on top of the smaller dish within the acrylic tank.

The skeletal microstructures were examined using scanning electron microscopy (SEM) (JSM-IT100, JEOL, Japan). For the SEM observations, polished sections of the skeletons were etched in a 0.1% formic acid solution for 20–40 s and then rinsed with distilled water and subjected to ultrasonic cleaning for 10–20 s. Next, the samples were mounted on stubs using double-sided adhesive tape and coated with a conductive platinum film using a sputter coater (JFC–1600, JEOL, Japan). The terminology of skeletal microstructural characteristics (e.g., rapid accretion deposits and thickening deposits) follows that of Stolarski, Brahmi et al., and Janiszewska et al.^17,18,24^.

X-ray CT imaging was conducted for morphological analysis using an inspeXio SMX–225CT micro focus X-ray CT system (Shimadzu, Japan) at the Tottori Institute of Industrial Technology, Tottori, Japan. Each *Heterocyathus* specimen was placed in a 2-mL polypropylene microtube (GDMST-2ML, As-One, Japan) and secured in the middle using melamine foam (GEKIOCHI-KUN, LEC, Inc., Japan). The capped tube was then fixed on the stage using curing tape. The specimens were scanned using the microfocal subsystem with X-ray settings at 80 kV and 40 μA. The major axes of the specimens were carefully aligned with the rotational axis of the sample stage. During each scan, a series of 2048 × 2048-pixel grayscale images were captured, representing cross-sectional views of the specimen perpendicular to the rotation axis. These images were subsequently imported into the InspeXio64 software for visualization of the 2D cross-sections and reconstruction of 3D images. After isolating the coral skeleton from the X-ray CT images, 3D models were reconstructed to analyse the orifice morphologies. The CT image data were processed and analysed using Slice^28^ via Cygwin on Windows 10, as well as ImageJ Fiji^29^ and Paint 3D (Microsoft). The grey-scale CT images of *Heterocyathus* were processed using the SlicePVR program within the Slice software. To extract the coral outline, the images were binarized, with pixel values ranging from 11 to 255 being replaced with 0, and pixel values from 1 to 10 being replaced with 1. Using Paint 3D, the non-outline portion of the image was erased, focusing solely on the orifice and pore outlines. The image sequence containing these outlines was further binarized using the SlicePVR program, replacing 0 pixel values with 1 and 1 pixel values with 0. Finally, the image sequence was reconstructed into a 3D image—utilized for morphological analysis—using the 3D viewer plugin in ImageJ Fiji.

### Supplementary Information

Supplementary Legends.

Supplementary Video 1.

## Data Availability

All data generated or analysed during this study are included in this published article.
