# Supplementary material for: Influence of sipunculan (peanut worm) activity on orifice formation in scleractinian Heterocyathus for adaptation to soft substrates
Source: Sci Rep. 2024 Apr 29;14:9817. doi: 10.1038/s41598-023-49631-y (PMC11059395; doi:10.1038/s41598-023-49631-y)
Supplement: Supplementary file 1 — Supplementary Legends. [file 41598_2023_49631_MOESM1_ESM.docx]

Supplementary Movie S1. Time-lapse movie of sipunculan excretion.
